# Integrating genetic ancestry into clinical care: Accuracy, utility, and stakeholder views

**DOI:** 10.1007/s12687-026-00880-0

**Published:** 2026-04-01

**Authors:** Jessica Prettyman, Thomas J Hoffmann, Sawona Biswas, Aleksandar Rajkovic

**Affiliations:** 1 Genetic Counseling Program, University of California, San Francisco, San Francisco, CA USA; 2 Institute for Human Genetics, University of California, San Francisco, San Francisco, CA USA; 3 Department of Pediatrics, University of California, San Francisco, San Francisco, CA USA; 4 Department of Epidemiology and Biostatistics, University of California, San Francisco, San Francisco, CA USA; 5 Department of Pathology, University of California, San Francisco, San Francisco, CA USA; 6 Department of Obstetrics and Gynecology, University of California, San Francisco, San Francisco, CA USA

**Keywords:** Genetic ancestry, Self‑identified race/ethnicity (SIRE), Electronic health record (EHR), Concordance analysis, Community genetics

## Abstract

How to integrate genetic ancestry into clinical care remains unsettled. We assessed (i) agreement between two commercial ancestry providers, (ii) concordance between multiple ancestry algorithms and self‑identified race/ethnicity (SIRE) recorded in the electronic health record (EHR), and (iii) participant perspectives on accuracy, clinical utility, discrimination risk, and EHR integration. Using data from 451 adults in the UCSF 3D Health Study, we harmonized ancestry outputs to five continental categories and quantified cross‑provider agreement and ancestry–SIRE concordance at ≥ 50%, ≥ 75%, and ≥ 90% thresholds; we also analyzed survey responses from 166 participants. Mean agreement between the two commercial providers was 58.4% overall (95% BCa CI: 57.4–59.4%), differing by ancestry: 54.7% European, 73.1% Asian, 57.6% Mixed American, and 76.2% African (overall *p* = 3.2 × 10⁻²⁰). At ≥ 90% concordance with SIRE, algorithms varied substantially: 2.6% for Ancestry 1 (11/428), 80.1% for Ancestry 2 (353/441), 88.0% for Ancestry 3 (388/441), and 89.3% for Ancestry 4 (394/441). When stratified by SIRE group, European and Asian participants showed consistently high concordance for Ancestries 2–4, while African and Mixed American subgroups showed lower and more variable concordance across all methods. Surveyed participants were largely nondistressed (only 1.2% reported distress); 51.2% agreed ancestry reports are accurate and 50.0% agreed ancestry could support more personalized care, while 52.4% anticipated discrimination. Opinions on storing ancestry in the EHR were mixed (agree: 38.6%; disagree: 30.1%; neutral: 28.9%). Findings show vendor/algorithm choice strongly shapes ancestry outputs and alignment with SIRE. Community genetics programs should avoid treating ancestry as a proxy for race, adopt vendor‑aware workflows, and implement transparent consent and safeguards before linking ancestry to the EHR.

**Supplementary Information:**

The online version contains supplementary material available at 10.1007/s12687-026-00880-0.

## Introduction

The use of race and genetic ancestry in clinical care has been a topic of intense debate in recent years (Arpone et al. 2024; Borrell et al. 2021; Cerdeña et al. 2022; Flanagin et al. 2021; Jorde and Bamshad 2020; Mauro et al. 2022; Redman et al. 2024; Roth et al. 2020; Sirugo et al. 2019; Vyas et al. 2020). The struggle with the use of race and genetic ancestry in clinical care is stated well in a perspective piece by Oberlander et al., who present a paradox that evolutionary geneticist Theodosius Dobzhansky struggled with of race which is “both believing race to be a tool to elucidate human genetic diversity and believing that race is a poorly defined marker of that diversity and an imprecise proxy for the relation between ancestry and genetics” (Oberlander et al., 2020). Race is a social construct that does not directly correspond to biology or genetics and provides limited information about an individual’s potential genetic predispositions (Borrell et al. 2021). In contrast, genetic ancestry utilizes genetic information to identify populations sharing common genetic variations, which can play a role in understanding an individual’s risk for various health conditions (Borrell et al. 2021; Elliott et al. 2023; Flanagin et al. 2021; Hsu et al. 2021; Jorde and Bamshad 2020). Importantly, human genetic diversity is not naturally partitioned into discrete groups; rather, genetic variation exists along a continuum and any clustering into categories depends on reference panels and analytic choices (Ding et al. 2023). Genetic ancestry reporting utilizes geographic groupings that may appear to reflect racial and ethnic groupings to identify commonalities and differences between populations. The estimation of genetic ancestry relies on current geographic labels, one’s interpretation of world geography, and colonialism (Bryc et al. 2015; Jorde and Bamshad 2020; Royal et al. 2010). It is important to note that genetic ancestry, like race, is a proxy measurement and can vary based on the parameters used; therefore, it can have similar limitations to race (Oberlander et al., 2020).

Currently, self-identified race and ethnicity (SIRE) is the most commonly used proxy for genetic ancestry in healthcare. It is critical to recognize that while genetic ancestry and SIRE are often used in tandem in healthcare, they measure distinct aspects of identity and health. Genetic ancestry reflects population-level genetic variation, whereas SIRE primarily reflects sociocultural factors. Genetic testing providers develop their ancestry tests independently, thus leading to potential inconsistencies among reports from different companies (Huml et al. 2020; Jorde and Bamshad 2020; Soundararajan et al. 2016). These inconsistencies are important to understand because they lessen the potential reliability of these tests and can cause confusion among healthcare providers and consumers. Limited research has investigated the concordance of genetic ancestry tests, with existing studies having limitations in sample size, diversity, and the number of commercial providers evaluated (Banda et al. 2015; Dumitrescu et al. 2010; Hall et al. 2014; Huml et al. 2020; Y. L. Lee et al. 2010). There is a need for further research to better characterize the discordances between genetic testing providers and SIRE.

In addition to direct-to-consumer and other commercial vendors, genetic ancestry is frequently inferred in-house in EHR-linked biobanks and health system genomic cohorts to support genetic association studies and downstream clinical translation. For example, the UCLA ATLAS Community Health Initiative characterized continental and subcontinental diversity using genetic data linked to the EHR and highlighted that genetically inferred ancestry and SIRE capture related but distinct information (Johnson et al. 2022). This biobank literature helps contextualize the present study, which examines concordance across multiple genetic ancestry estimation approaches and their relationship to EHR-recorded SIRE and participant perspectives.

Establishing the clinical utility of genetic ancestry information is crucial for its potential integration into the electronic health record (EHR). It is essential to understand if providers would find this information valuable, if they would use it in clinical decision-making, and if they trust the validity of the data. Currently, there is minimal research into the utility of genetic ancestry in clinical care, and it is unknown if the perceived utility will increase if genetic ancestry testing becomes more common in clinical care. Studies exploring provider perspectives on genetic ancestry testing are also limited, with no research examining their views on its potential utility in the EHR (Haga et al. 2019; Hubbel et al. 2023; Kirkpatrick and Rashkin 2017; Nelson et al. 2018). Literature on patient perspectives has found mixed opinions on genetic ancestry testing and its impact on personal identity (Blanchard et al. 2019; S. S.-J. Lee 2013; Ruhl et al. 2020; Shim et al. 2018). Some individuals feel as though genetic ancestry has an impact on identity, and if it is different from their perceived ancestry, it can lead to a reorganization of their understanding of themselves. Others feel as though their genetic ancestry is separate from their identity.

The increasing availability of genetic testing is driving a growing interest in personalized medicine (Tiner et al. 2022). Genetic ancestry information has been shown to offer insights into an individual’s personalized risk for having or developing monogenic, polygenic, and multifactorial conditions (Cappetta et al. 2015; Check et al. 2022; Mosley et al. 2020). However, the validity and clinical impact of these tests are still under investigation, primarily due to the limited availability of diverse genetic data. As the use of these tests expands and their utility improves, genetic counselors will play a crucial role in interpreting and communicating these results to patients and non-genetic providers.

This study aimed to explore the utility and implications of genetic ancestry data through the perspectives of participants in the UCSF 3D Health Study. The 3D Health Study, launched in 2019, investigates the predictive value of genome sequencing (GS) in a healthy adult population. Understanding the current limitations of genetic ancestry reports and participant perspectives on their clinical and personal utility is essential for informing the responsible integration of this information into healthcare.

## Materials and methods

This project consisted of two separate analyses, a statistical analysis of genetic ancestry data and an analysis of participant surveys.

### Institutional review board approval

This project was performed with approval from the University of California, San Francisco (UCSF) Institutional Review Board (IRB). Modifications were made to the existing 3D Health Study protocol to distribute the additional surveys and perform the secondary concordance analysis. The 3D Health Study at the University of California, San Francisco (UCSF) was launched in 2019 as a pilot project to investigate the predictive value of genome sequencing (GS) in a healthy adult population. With an enrollment of 535 participants, the study aims to establish institutional best practices for obtaining, storing, and communicating genomic information. In addition to understanding the utility of WGS for healthy adults, the study focuses on determining the types of genomic results participants want to receive.

### Genetic ancestry concordance analysis

Genetic ancestry reports were collected from two commercial genetic testing companies, Ancestry 1 and Ancestry 2. In addition, two supplemental genetic ancestry calculations, Ancestry 3 and Ancestry 4, were conducted by our team using publicly available reference panels from the 1,000 Genomes Project and the 100,000 Genomes Project (Auton et al. 2015; Turnbull et al. 2018). Because the underlying ancestry inference algorithms and reference panel choices for commercial vendors are not fully disclosed, we treated Ancestry 1 and Ancestry 2 as black-box outputs and compared their reported continental ancestry proportions. The participant-level ancestry proportions produced from all four sources were analyzed for concordance. Missing data for any ancestry reports were excluded from analyses where no clear imputation could be performed. Unless otherwise noted or performed by the commercial genetic testing companies, analyses were conducted using R v4.1.0 (R Foundation for Statistical Computing, 2014). Confidence intervals for mean concordance estimates in Table 1 were computed using nonparametric bootstrap resampling (1,000 replicates) with bias-corrected and accelerated (BCa) intervals. An additional concordance analysis was performed comparing the genetic ancestry reports to SIRE data collected from Apex, UCSF’s electronic health record system.

**Table 1:** Concordance between genetic ancestry calculations. Genetic ancestry results from two different commercial genetic testing providers were compared for 451 samples in the UCSF 3D Health Study. The percent of overlapping ancestry determined the agreement from each ancestry location (European, African (Afr), Asian (As), and Mixed American (Amr)). P-values were determined using a Wilcoxon Rank Sum test comparing each pairwise group. Confidence intervals were estimated from nonparametric bootstrap (1,000 replicates) bias-corrected and accelerated (BCa) confidence interval

Ancestry 1 vs. Ancestry 2 (*n* = 451)	Min	Median	Max	Mean (95% CI)		*P*-values	
Genetic Ancestry					Afr	As	Amr
**Overall**	0.00%	56.16%	94.96%	58.41% (57.40%, 59.43%)			
European (*n* = 326)	26.89%	54.63%	78.03%	54.73% (53.94%, 55.45%)	8.2e-5**	6.5e-19**	0.016*
African (*n* = 9)	52.20%	79.99%	94.96%	76.21% (65.48%, 84.56%)		0.37	0.017*
Asian (*n* = 73)	31.42%	78.45%	89.94%	73.07% (69.24%, 75.87%)			2.0e-4**
Mixed American (*n* = 18)	0.00%	61.96%	80.96%	57.55% (46.27%, 63.16%)			

*P < 0.05; **P < 0.005

Ancestry 3 and Ancestry 4 (in-house analyses): To support transparency and reproducibility, we expanded the Methods and Supplemental Materials to describe the pipeline used to generate Ancestry 3 and Ancestry 4, including genotype quality control, reference panel selection, and model/parameter choices. Briefly, ancestry proportions were estimated using a reference-based approach that produces per-individual continental ancestry proportions aligned to the broad categories reported by commercial vendors (African, Mixed American/Admixed American, Asian, and European; with South Asian and East Asian components combined as Asian for downstream comparisons).

Specifically, we (i) restricted analyses to autosomal bi-allelic single-nucleotide variants present in both the participant data and the chosen reference panel; (ii) harmonized alleles/strands and performed standard genotype quality control; (iii) pruned variants for linkage disequilibrium to reduce redundancy; and (iv) estimated ancestry proportions using a supervised reference-based model (e.g., principal component projection followed by ancestry proportion estimation or a model-based clustering approach). For Ancestry 3, the reference panel was derived from 1,000 Genomes continental group labels (AFR/AMR/EAS/EUR/SAS). For Ancestry 4, the reference panel was derived from the 100,000 Genomes Project. Full software names, versions, and parameter values will be provided in the public code repository upon release.

The first method of analysis, which compared the two commercial genetic testing companies and two additional genetic ancestry analyses, was developed from a prior study on genetic ancestry data from direct-to-consumer companies (Rubanovich et al. 2021). We looked for the overall concordance in genetic ancestries reported by the four genetic ancestry analyses to quantify the variation in results for each participant. The companies reported genetic ancestry at different geographical levels. Ancestry 1 determines genetic ancestry at a more specific geographic location. Ancestry 2 reports genetic ancestry for five geographic regions; African, Mixed American, East Asian, European, and South Asian. For the analysis, we grouped the reported ancestries for Ancestry 1 into the five categories determined by Ancestry 2 to run the comparison (Supplemental Table 1). Participants were reported to have genetic ancestry from five geographic regions including African (AFR), Mixed American (AMR), East Asian (EAS), European (EUR), and South Asian (SAS). The additional genetic ancestry calculations performed by our team reported genetic ancestry in the same five geographic regions that the commercial genetic ancestries were grouped in. The concordance analysis aimed to determine the percent agreement between the reports for each participant and then determine the overall agreement when comparing each report to one another. The analysis performed on the data is below.

$$\begin{array}{c}Participant\;Percent\;Agreement\;=\;(lower\;AFR\;ancestry\;percentage)\;+\\\;(lower\;AMR\;ancestry\;percentage)\;+\;(lower\;EAS\;ancestry\;percentage)\;\\+\;(lower\;EUR\;ancestry\;percentage)\;+\;(lower\;SAS\;ancestry\;percentage)\\Total\;agreement\;={\textstyle\sum_{}}i\;Participant\;i\;Percent\;Agreement\end{array}\\$$

The agreements between the providers as well as between different ancestry groups were then compared. A Kruskal-Wallis test was used to determine if there were statistically significant differences between genetic ancestry groups. Pair-wise comparisons were run using a Wilcoxon Rank Sum test to identify statistically significant differences when comparing the mean concordance of ancestry groups.

The second analysis compared the genetic ancestry data to self-identified race and ethnicity gathered from the UCSF electronic health record (EHR) system, Apex. The self-identified race and ethnicity (SIRE) was gathered for each participant in the 3D Health Study. UCSF’s EHR system, Apex, includes seven options for race/ethnicity including Hispanic or Latino, Black or African American, White/Caucasian, Asian, Native Hawaiian or Other Pacific Islander, American Indian or Alaska Native, and Mixed. More than one race/ethnicity can be selected within the UCSF EHR. For the analysis, these race/ethnicity options were harmonized into broad categories that approximated the continental groupings used in the genetic ancestry reports: Asian (AS), African (AFR), Mixed American (AMR), European (EUR), and no data (ND). Asian included those whose Apex-reported race was Asian. African included those who reported Black or African American. Mixed American included those whose race/ethnicity was Hispanic or Latino and/or American Indian or Alaska Native. Those who selected Mixed were placed into ND because their race could not be directly evaluated in this concordance framework. Individuals who selected Native Hawaiian or Other Pacific Islander were not grouped with Asian; because the commercial ancestry reports used in this study did not report a directly comparable Oceanian/Pacific Islander component, these participants were categorized as ND for the primary SIRE concordance analysis. Individuals with only one race/ethnicity selected were treated as 100% of that race/ethnicity. Individuals with more than one race/ethnicity (other than Mixed) selected were represented as equal fractions across the selected categories (e.g., Asian and European would be 50% Asian and 50% European) to enable a proportion-based concordance metric. We acknowledge that this simplification does not represent true genetic ancestry distributions and may bias concordance estimates; we discuss this as a limitation. The genetic ancestry data were then compared to the SIRE of the participants in the UCSF 3D Health Study. The methods of the analysis were based on prior work that analyzed concordance between SIRE and genetic ancestry for those of European or African ancestry (Dumitrescu et al. 2010). Specifically, we reported the percentage of ancestry reports that met or exceeded each of three thresholds (T) of concordance to SIRE (T = 50%, 75%, and 90%). The percentage of concordant ancestries at each threshold was then compared for each ancestry provider using a matrix of McNemar’s test to identify statistically significant differences between results.

### 3D health participant survey

The 3D Health Participant Survey aimed to gather patients’ perspectives on genetic ancestry testing, its perceived benefits, drawbacks, and impact on identity. The participant survey was adapted from validated instruments in previous studies on genetic ancestry testing (Rubanovich et al. 2021). The survey questions were pre-tested for clarity, and measures such as Likert scales were used to assess the range of participant perspectives (Supplemental Materials). The questionnaire was sent to 527 active 3D Health participants who had not withdrawn, been lost to follow-up, or died since enrolling. An initial email asked about their interest in receiving genetic ancestry results and informed them about the additional questionnaire. A total of 277 interested participants received their results from both commercial genetic ancestry providers and the questionnaire, to be completed after viewing the results. Quantitative and qualitative metrics were used to analyze responses. Descriptive statistics summarized demographics, perspectives on EHR integration, utility, impact on identity, and common concerns/benefits. Fisher’s exact tests were used to compare response distributions across self-identified race/ethnicity groups; given small cell counts in some groups, these comparisons are interpreted cautiously and are presented primarily as exploratory.

## Results

Data for this study were collected from the UCSF 3D Health Study, which aims to investigate the predictive value of whole genome sequencing in a healthy adult population and to establish best practices for obtaining, storing, and communicating genomic information.

### Concordance analysis

The concordance analysis utilized genetic data from 451 participants in the UCSF 3D Health Study to compare genetic ancestry results from two commercial genetic testing companies and a 3rd-party analysis, performed with publicly available genetic databases. The analysis aimed to determine the concordance between the different genetic ancestry calculations and to compare genetic ancestry results with self-identified race and ethnicity (SIRE) data.

#### Concordance between commercial genetic ancestry companies

**Fig. 1:**
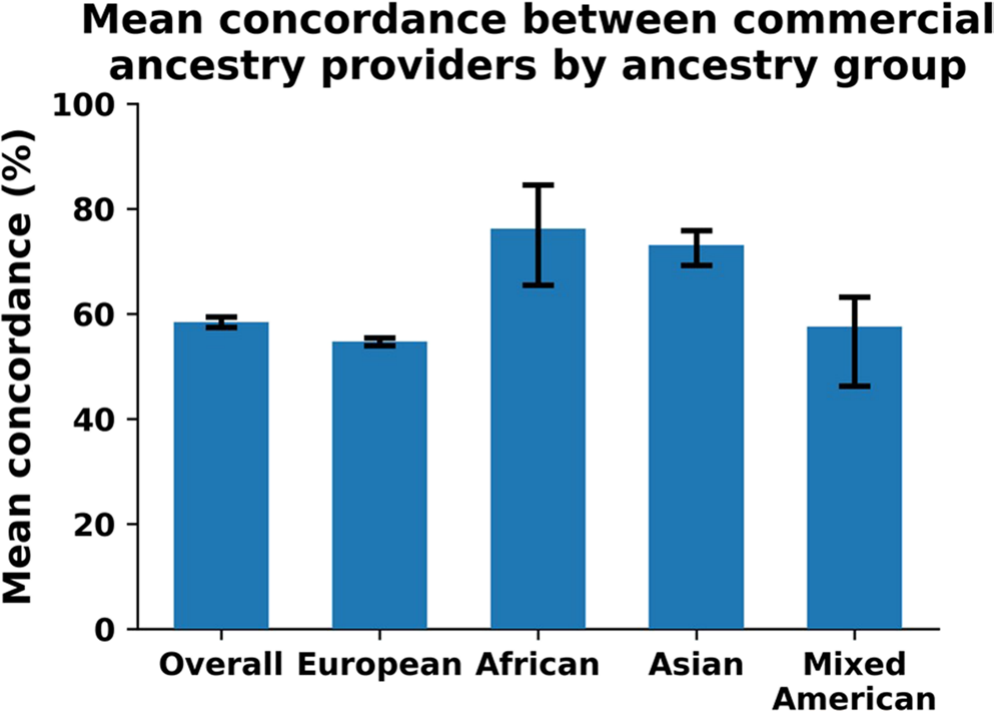
Mean concordance (%) between two commercial genetic ancestry providers (Ancestry 1 vs. Ancestry 2), overall and by ancestry group. Error bars represent 95% BCa bootstrap confidence intervals

The concordance analysis compared the genetic ancestry results from two different commercial genetic testing companies, referred to as Ancestry 1 and Ancestry 2, for 451 samples in the UCSF 3D Health Study. The overall mean concordance between Ancestry 1 and Ancestry 2 was 58.41% (95% BCa CI: 57.40–59.43%), with a median of 56.16% and a range of 0.00–94.96% (Table 1). Mean concordance varied across genetic ancestry groups: European participants (*n* = 326) had the lowest mean concordance at 54.73% (95% BCa CI: 53.94–55.45%), while African participants (*n* = 9) had the highest at 76.21% (95% BCa CI: 65.48–84.56%). Asian participants (*n* = 73) had a mean concordance of 73.07% (95% BCa CI: 69.24–75.87%), and Mixed American participants (*n* = 18) had a mean of 57.55% (95% BCa CI: 46.27–63.16%). The wider confidence intervals for the African and Mixed American subgroups reflect the greater uncertainty associated with their smaller sample sizes. The minimum concordance observed was 0% (Table 1), indicating that a small number of participants received non-overlapping continental ancestry assignments across vendors; we discuss potential sources of these extreme discrepancies, including differences in reference panels, category definitions, and handling of low-level ancestry components. A significant difference was found when comparing the overall mean concordance by ancestry (p-value = 3.2e-20). The pair-wise comparison found statistically significant differences between all of the ancestry groups except when Asian and African groups were compared (Table 1). These results are summarized visually in Fig. 1.

#### Concordance of SIRE and genetic ancestry

In this analysis, the genetic ancestry results from four different sources (two commercial genetic testing companies, Ancestry 1 and Ancestry 2, and two supplemental ancestry calculations from publicly available genetic databases, Ancestry 3 and Ancestry 4) were compared to the SIRE for samples from the UCSF 3D Health study, gathered from the UCSF EHR system, Apex.

**Fig. 2:**
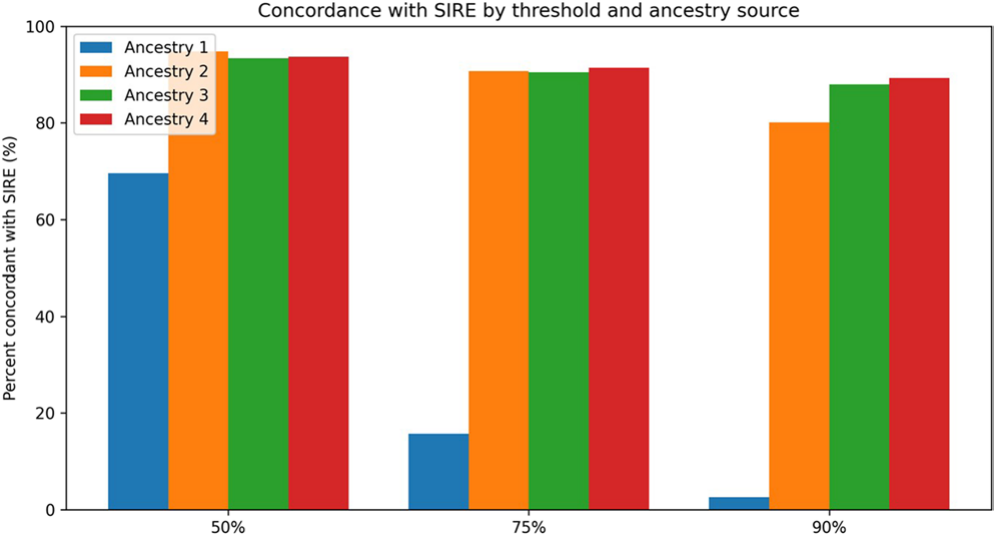
Percent concordance between each ancestry source and EHR-recorded self-identified race and ethnicity (SIRE) at ≥ 50%, ≥ 75%, and ≥ 90% thresholds

Ancestry 1 was found to have the lowest concordance with SIRE across all thresholds: 69.63% of samples had at least a 50% concordance, 15.65% had at least a 75% concordance, and 2.57% had at least a 90% concordance with SIRE (Table 2). Ancestry 2 had a substantially higher concordance with SIRE: 94.78% at ≥ 50%, 90.70% at ≥ 75%, and 80.05% at ≥ 90% (Table 2). Ancestry 3 and Ancestry 4 had the highest concordance with SIRE among the four genetic ancestry sources: for Ancestry 3, 87.98% of samples were at least 90% concordant with SIRE, while for Ancestry 4, 89.34% were at least 90% concordant (Table 2). These threshold concordance patterns are summarized in Fig. 2.

**Table 2:** Concordance between genetic ancestry results and Apex-reported race. Genetic ancestry results from two commercial providers and two supplemental ancestry calculations from a 3rd-party software were compared to Apex-reported race/ethnicity for samples from the UCSF 3D Health study. Results are stratified by SIRE group within each ancestry calculation

Genetic Ancestry Calculation	≥ 50%	≥ 75%	≥ 90%
**Ancestry 1**	69.63% (298/428)	15.65% (67/428)	2.57% (11/428)
*European*	67.48% (220/326)	0% (0/326)	0% (0/326)
*African*	77.78% (7/9)	44.44% (4/9)	22.22% (2/9)
*Asian*	93.15% (68/73)	86.30% (63/73)	12.33% (9/73)
*Mixed American*	16.67% (3/18)	0.00% (0/18)	0.00% (0/18)
**Ancestry 2**	94.78% (418/441)	90.70% (400/441)	80.05% (353/441)
*European*	97.92% (330/337)	96.14% (324/337)	83.38% (281/337)
*African*	66.67% (6/9)	55.56% (5/9)	33.33% (3/9)
*Asian*	95.95% (71/74)	90.54% (67/74)	87.84% (65/74)
*Mixed American*	55.56% (10/18)	16.67% (3/18)	16.67% (3/18)
**Ancestry 3**	93.42% (412/441)	90.48% (399/441)	87.98% (388/441)
*European*	98.51% (332/337)	96.44% (325/337)	94.96% (320/337)
*African*	66.67% (6/9)	55.56% (5/9)	22.22% (2/9)
*Asian*	98.65% (73/74)	91.89% (68/74)	87.84% (65/74)
*Mixed American*	0.00% (0/18)	0.00% (0/18)	0.00% (0/18)
**Ancestry 4**	93.65% (413/441)	91.38% (403/441)	89.34% (394/441)
*European*	98.81% (333/337)	97.33% (328/337)	96.74% (326/337)
*African*	66.67% (6/9)	55.56% (5/9)	22.22% (2/9)
*Asian*	94.59% (70/74)	91.89% (68/74)	87.84% (65/74)
*Mixed American*	16.67% (3/18)	5.56% (1/18)	0.00% (0/18)

When concordance was stratified by SIRE group (Table 2; Supplemental Table 8), the overall pattern of Ancestry 1 showing lower concordance and Ancestries 2–4 showing higher concordance was generally consistent across SIRE categories. European participants consistently showed high concordance for Ancestries 2–4 (83–97% at the ≥ 90% threshold) but markedly lower concordance for Ancestry 1 (0% at ≥ 75%). Asian participants showed strong concordance across all four sources at the ≥ 75% threshold (86–92%) but lower concordance for Ancestry 1 at ≥ 90% (12.33% vs. 87–88% for Ancestries 2–4). African participants (*n* = 9) showed moderate concordance across all sources (55–78% at ≥ 50%; 44–56% at ≥ 75%), with the small sample size limiting precision. Mixed American participants (*n* = 18) showed the greatest variability across methods, with concordance at the ≥ 50% threshold ranging from 0% (Ancestry 3) to 55.56% (Ancestry 2), underscoring the difficulty of capturing admixed genetic ancestry with proportion-based concordance measures. Small subgroup sizes for African and Mixed American participants limit interpretation of within-group differences.

Pair-wise comparisons of the ancestry groups found a statistically significant difference in the concordance between Ancestry 1 and every other ancestry (Ancestry 2, Ancestry 3, Ancestry 4) at the 50% threshold (p-value = 5.0e-24; 1.4e-21; 1.5e-22) and 75% threshold (p-value = 1.7e-70; 6.4e-71; 1.4e-71) (Supplemental Table 5). There was a significant difference between all of the ancestries at the 90% threshold (Supplemental Table 5).

### Participant survey responses

#### Respondent demographics

We received a total of 166 responses from 3D Health Study participants, a 59.9% response rate. The majority of the respondents to the study identified as women (60.8%), 60 or above (49.4%), and white (74.7%). Participants identifying as Asian represented 20.5% of the respondents, while 5.4% identified as Hispanic/Latino, 2.4% as Black/African American, and 0.6% as American Indian/Alaska Native (Supplemental Table 2).

#### Impact of genetic ancestry on identity

Participants were asked about their reactions to their genetic ancestry results, specifically whether the results were surprising or unexpected. The responses were evenly distributed, with 31.9% answering “Yes,” 31.3% answering “No,” and 35.5% answering “Somewhat/Maybe” (Table 3). When responses were stratified by SIRE (Supplemental Table 7), 51.7% of those identifying as Asian responded “No,” indicating that their genetic ancestry results were not surprising or unexpected. In contrast, 100% of those identifying as African, 50% of those identifying as Mixed American, and 61.5% of those identifying as Other selected that their genetic ancestry results were somewhat or maybe surprising or unexpected. The differences in responses across SIRE groups were statistically significant (p-value = 0.0065) (Table 3).

**Table 3:** **3D **Health Study participant responses to questions regarding the impact of genetic ancestry results on identity. Responses represent overall totals from all participants (*N* = 166). Questions based on Rubanovich et al. 2021. SIRE-stratified responses are provided in Supplemental Table 7. P-values were determined using Fisher’s Exact test comparing response distributions across SIRE groups

Domain	Question	Response 1	Response 2	Response 3	Response 4	*p*-value (SIRE)
*General* *Response*	Were your ancestry test results surprising or unexpected?	Yes 53 (31.9%)	No 52 (31.3%)	Somewhat/Maybe 59 (35.5%)	Missing 2 (1.2%)	0.0065*
*General* *Response*	Were your ancestry test results undesired or distressing?	Yes 2 (1.2%)	No 158 (95.2%)	Somewhat/Maybe 3 (1.8%)	Missing 3 (1.8%)	0.72
*Cultural/* *Personal* *Identity*	Do your ancestry test results change your perceptions of your cultural roots?	Yes 18 (10.8%)	No 109 (65.7%)	Somewhat/Maybe 36 (21.7%)	Missing 3 (1.8%)	0.076
*Cultural/* *Personal* *Identity*	Do your ancestry test results change the likelihood that you would travel to certain parts of the world?	Yes 5 (3.0%)	No 148 (89.2%)	Somewhat/Maybe 9 (5.4%)	Missing 4 (2.4%)	0.57
*Cultural/* *Personal* *Identity*	Do your ancestry test results change how you view certain cultures or world regions?	Yes 4 (2.4%)	No 154 (92.8%)	Somewhat/Maybe 4 (2.4%)	Missing 4 (2.4%)	0.13
*Cultural/* *Personal* *Identity*	Would you say your ancestry test results have reshaped your personal identity?	Yes 7 (4.2%)	No 141 (84.9%)	Somewhat/Maybe 14 (8.4%)	Missing 4 (2.4%)	0.096
*Sharing* *Results*	Do you plan to share your ancestry test results with your family members?	Yes 122 (73.5%)	No 20 (12.0%)	Somewhat/Maybe 22 (13.3%)	Missing 2 (1.2%)	0.33
*Sharing* *Results*	Will you provide or discuss your ancestry test results with your physician or a healthcare provider?	Yes 34 (20.5%)	No 70 (42.2%)	Somewhat/Maybe 59 (35.5%)	Missing 3 (1.8%)	0.061
*Ancestry* *Testing*	If you had genetic ancestry testing previously, do you perceive your current results to be different?	Yes 37 (22.3%)	No 26 (15.7%)	Somewhat 18 (10.8%)	N/A / Missing 73 (44.0%) / 12 (7.2%)	0.0066*
*Ancestry* *Testing*	Does the experience of undergoing genetic ancestry testing make you more or less likely to have other genetic tests?	More Likely 53 (31.9%)	Less Likely 16 (9.6%)	No Change 94 (56.6%)	Missing 3 (1.8%)	0.016*

*P < 0.05. SIRE-stratified responses by question are provided in Supplemental Table 7

Participants were also asked if their genetic ancestry reports were undesired/distressing, changed their perceptions of cultural identity, or reshaped their identity. The majority of respondents selected “No” for all three questions (95.2%, 65.7%, and 84.9%, respectively) (Table 3).

For respondents who had undergone genetic ancestry testing prior to the study, there was a mixed response regarding whether they perceived their current genetic ancestry results as different from their previous results (Table 3).

Participants were asked if undergoing genetic ancestry testing changed the likelihood of them having other genetic tests in the future. 56.6% of respondents felt no change in the likelihood, but there was a statistically significant difference in responses across SIRE groups (p-value = 0.016; Table 3; Supplemental Table 7). The majority of White/European (55.7%), Asian (65.5%), Mixed American (50%), and Other (61.5%) respondents felt there was no change, while all respondents who identified as Black/African American (100%) felt they were more likely to have genetic ancestry testing in the future. It is important to note that there were only a total of 3 responses from those that identified as Black/African American; the small sample size may be the reason for the skew in the responses.

#### Perceived accuracy and utility of genetic ancestry

Participants were asked about their perceptions of the accuracy and utility of genetic ancestry reports. 44.6% of 3D Health participants somewhat agreed and 6.6% strongly agreed that genetic ancestry reports are accurate (Table 4). When asked if genetic ancestry reports will allow for more personalized care, the majority of respondents selected neutral (30.1%), somewhat agree (33.7%), or strongly agree (16.3%).

**Table 4:** Participant responses to questions regarding genetic ancestry testing accuracy and utility. Responses were totaled for the overall responses from all participants and then calculated based on the participant’s self-identified race/ethnicity. A Fisher’s Exact test was used to determine if there was a statistically significant difference in responses between ancestry groups

Question	Strongly Disagree	Somewhat Disagree	Neutral	Somewhat Agree	Strongly Agree	Missing
Genetic ancestry reports are accurate.	14 (8.4%)	22 (13.3%)	42 (25.3%)	74 (44.6%)	11 (6.6%)	3 (1.8%)
Genetic ancestry reports will allow for more personalized care.	4 (2.4%)	23 (13.9%)	50 (30.1%)	56 (33.7%)	27 (16.3%)	6 (3.6%)
Genetic ancestry reports should be used by healthcare providers to make health related decisions.	11 (6.6%)	21 (12.7%)	54 (32.5%)	57 (34.3%)	20 (12.0%)	3 (1.8%)
Genetic ancestry reports should be used by healthcare providers to better understand their patients as a whole.	8 (4.8%)	17 (10.2%)	43 (25.9%)	72 (43.4%)	23 (13.9%)	3 (1.8%)
Genetic ancestry reports should be in the electronic health record.	14 (8.4%)	36 (21.7%)	48 (28.9%)	38 (22.9%)	26 (15.7%)	4 (2.4%)

Overall responses only. SIRE-stratified responses are available in the original Table 4format and by request. *P < 0.05

Participants were also asked if genetic ancestry reports should be used by healthcare providers to make health-related decisions and to better understand their patients as a whole. Overall, 78.8% of respondents were neutral, somewhat agree, or strongly agree that genetic ancestry reports should be used for health-related decisions, and 83.2% were neutral, somewhat agree, or strongly agree that providers could use genetic ancestry to better understand their patients (Table 4). There was a statistically significant difference in response distributions across SIRE groups (Fisher’s exact p-value = 0.035); however, this result should be interpreted cautiously given very small cell counts in some groups (e.g., Black/African American *n* = 3).

#### Perspectives on integration of genetic ancestry into the EHR

Participants were asked if genetic ancestry reports should be included in the electronic health record (EHR). Overall, 38.6% of respondents somewhat or strongly agreed with this idea, 30.1% somewhat or strongly disagreed, and 28.9% were neutral (Table 4). Participants were also asked about their concerns regarding the possible unintended outcomes of including genetic ancestry data in the EHR. The most commonly selected concerns were privacy (59.0%) and misuse of genetic data (74.1%) (Supplemental Fig. 2). Other concerns reported by participants included data breaches, unintentional biases, discordance between different ancestry reports, and questionable accuracy. When asked specifically about their thoughts on including genetic ancestry reports in the EHR, the majority of respondents selected privacy concerns (58.4%) and misuse of genetic data (69.9%) (Supplemental Fig. 3). Additional concerns included insurance companies using the results to determine risk for pre-existing conditions or risk factors, questionable accuracy, and data breaches.

#### Genetic discrimination

Participants were asked if they expected genetic ancestry reports to be used to discriminate between groups; 52.4% responded “Yes” and 42.2% responded “No” (Table 5). There was no statistically significant difference in the responses between different SIRE groups. All respondents identifying as Black/African American responded “No,” while Mixed Americans had the highest percentage of “Yes” responses (66.7%) (Table 5).

**Table 5:** 3D Health participant responses to concerns about genetic ancestry being used to discriminate between groups

Do you expect genetic ancestry reports will be used to discriminate?	Yes	No	Missing	*P*-Value
**Overall**	87 (52.4%)	70 (42.2%)	9 (5.4%)	
White/European (*n* = 115)	58 (50.4%)	49 (42.6%)	8 (7.0%)	
Black/African American (*n* = 3)	0 (0.0%)	3 (100.0%)	0 (0.0%)	
Asian (*n* = 29)	17 (58.6%)	11 (37.9%)	1 (3.4%)	0.39
Mixed American (*n* = 6)	4 (66.7%)	2 (33.3%)	0 (0.0%)	
Other (*n* = 13)	8 (61.5%)	5 (38.5%)	0 (0.0%)	

## Discussion

Our study aimed to identify the concordance of genetic ancestry reports calculated by two commercial genetic testing companies and two 3rd-party analyses. Additionally, it sought to explore the perspectives of 3D Health Study participants on the utility and implications of genetic ancestry data.

The comparison of genetic ancestry results from two commercial genetic testing companies revealed an average concordance of only 58.4% (Table 1), indicating that individuals may receive notably different results depending on the genetic testing company. This finding aligns with prior studies that have found similar discordances between genetic ancestry reports (Huml et al. 2020). Despite both commercial genetic testing companies using the 1,000 Genomes Project database, the discrepancies in their reports demonstrate that genetic ancestry calculations can vary significantly, even when using the same reference population. Unfortunately, we were unable to gather additional information from either commercial genetic testing company on the intricacies of their genetic ancestry test and other proprietary data they may use to calculate genetic ancestry. These inconsistencies and lack of access to information on the internal testing being done underscore the need for standardization in the calculation of genetic ancestry before its potential integration into the EHR.

Participants identifying as White had the most discordant results between the two commercial genetic testing companies, with a mean concordance of 54.7% (Table 1). This finding was surprising given that individuals of White or European ancestry are the most represented in genetic research. We expected that those of non-White racial and ethnic groups would have more discordant findings given racial inequities in genetics research leading to a lack of diversity in the majority of genetic and medical research. This finding indicated that even for populations that have been used for much of the development of genetic testing there are still differences in interpretation of this data between genetic testing companies. It is likely that due to centuries of admixture as a result of colonialism, White individuals in the United States are more likely to not be 100% White or European (Bryc et al. 2015). Knowing that an individual’s SIRE is likely to not correspond directly to genetic ancestry is important for providers to be aware of. As the availability of larger genetic testing, exome and genome sequencing, there is less limitation in the forms of tests that are being ordered for individuals and therefore potentially a lesser need for targeted race-based genetic testing. Prenatal carrier screening has been a form of genetic testing that has previously focused on SIRE to determine the panel size and targets. A study in 2020 found that race-based prenatal carrier screening misses the identification of a large number of carriers because genetic variants are not limited to specific races or ethnicities (Kaseniit et al. 2020). Providers need to be aware of the benefits and limitations of the utility of SIRE and/or genetic ancestry in clinical care and be informed to make the correct clinical decisions based on this information.

Following distribution of genetic ancestry reports and completion of the survey, some participants contacted the study team with questions about report accuracy or whether they had received the correct report. Because these contacts were not systematically collected as an outcome measure, we report them here as qualitative feedback and as motivation for clearer communication around what genetic ancestry estimates can and cannot represent. In response to participant questions, we provided additional information about how genetic ancestry is estimated and how to interpret their results. Overall, survey responses suggested generally positive perceptions of genetic ancestry testing; for example, among White/European respondents, 77.4% were neutral or agreed that genetic ancestry reports are accurate (Table 4).

Previous studies have found that discrepancies between genetic ancestry and an individual’s perceived ancestry can lead to confusion and a need to reevaluate one’s identity (Banda et al. 2015; Y. L. Lee et al. 2010). However, individuals often prioritize their lived racial identity over reported genetic ancestry (Blanchard et al. 2019; Shim et al. 2018). The impact of genetic ancestry testing on patient identity was particularly noteworthy, as participants reported varying degrees of surprise and shifts in their perceptions of their cultural roots. Genetic counselors must be prepared to help patients navigate these complex emotional responses, particularly when ancestry results differ from self-perception.

Participants in the 3D Health Study were largely neutral or in agreement regarding the integration of genetic ancestry into the EHR (Table 4; Supplemental Table 6), possibly indicating a lack of established utility for genetic ancestry at this time. Concerns around data privacy and potential misuse were prominent among participants, with potential risks including data breaches, misuse of ancestry data for insurance purposes, and inappropriate application of genetic ancestry in clinical decision-making. These risks must be addressed with robust safeguards before integrating genetic ancestry into the EHR. Prior studies have found genetic ancestry to be important in interpreting polygenic risk scores for an individual’s risk of polygenic and multifactorial conditions (Borrell et al. 2021; Khera et al. 2018; Lambert et al. 2019). Certain SIRE have also been shown to correlate with a higher incidence of variants of uncertain significance (VUSs) in genetic testing, likely due to the underrepresentation of non-White racial and ethnic groups in genetic studies (Chen et al. 2023). Awareness of the increased likelihood of VUSs based on ancestry can inform pre-test genetic counseling and help manage patient expectations regarding uncertain results. A significant concern regarding the increased use of genetic ancestry in medical care is the potential conflation of genetic ancestry as the sole explanation for health disparities among different groups (Cerdeña et al. 2022; Roth et al. 2020). As research on the utility of genetic ancestry testing expands, it is crucial to consider other external factors that may contribute to differences in health risks and outcomes, such as social determinants of health (SDH) (Braveman and Gottlieb 2014).

A key limitation of this study is the relatively small sample size of non-European ancestry groups, which may have affected our ability to detect differences in concordance rates across ancestries. Minimal diversity in the study also likely impacted our ability to identify any significant differences in the perspectives of individuals within different SIRE groups. Further studies should include larger and more diverse populations to ensure broader applicability of the findings. Additionally, our grouping of genetic ancestries and SIRE may have introduced our own biases into the data analysis. Only having access to the SIRE grouping within the EHR system limited our ability to fully understand the proportion of different SIRE groups with which an individual identifies. For those with more than one SIRE group selected, we split their SIRE evenly, although this is likely not representative of their true SIRE breakdown. We acknowledge that this is a significant limitation of our analysis and introduces a bias in the dataset. We found that there was minimal interest in the introduction of genetic ancestry into the EHR, this may be due to the limited knowledge of the potential utility of genetic ancestry in clinical care. Lastly, the 3D Health participants were already enrolled in a research study, potentially biasing their responses in favor of genetic testing and its utility in the EHR.

Future studies on genetic ancestry testing should strive to include more diverse study populations to better identify inaccuracies in genetic ancestry reporting and determine best practices for calculating genetic ancestry. Moreover, further research is needed to investigate the potential utility of genetic ancestry data in clinical care algorithms and polygenic risk scores, which will help guide the integration of genetic ancestry into the EHR.

## Supplementary Information

Below is the link to the electronic supplementary material.

Supplementary Material 1

## Data Availability

The data analyzed in this study were generated as part of the UCSF 3D Health Study. De-identified genetic ancestry and survey datasets supporting the findings of this article are available from the corresponding author, Aleksandar Rajkovic (aleks.rajkovic@ucsf.edu), upon reasonable request and with approval from the UCSF Institutional Review Board, in accordance with participant consent and institutional data-sharing policies. No publicly archived datasets were used in this study.
