# Supplementary material for: Integrating genetic ancestry into clinical care: Accuracy, utility, and stakeholder views
Source: J Community Genet. 2026 Apr 1;17(2):46. doi: 10.1007/s12687-026-00880-0 (PMC13043983; doi:10.1007/s12687-026-00880-0)
Supplement: Supplementary file 1 — Supplementary Material 1 [file 12687_2026_880_MOESM1_ESM.docx]

**Supplemental Materials**

*Integrating Genetic Ancestry into Clinical Care: Accuracy, Utility, and Stakeholder Views*

Prettyman J, Hoffmann TJ, Biswas S, Rajkovic A

**Supplemental Table 1.**

Genetic ancestry reporting from each genetic ancestry provider and Apex-reported race/ethnicity. Ancestry 1 reports ancestry at a more specific geographic level; sub-categories were grouped into the five continental groupings used by Ancestry 2 for comparison.

| **Genetic Ancestry Grouping** | **Ancestry 1** | **Ancestry 2** | **Apex-Reported Race/Ethnicity** |
| --- | --- | --- | --- |
| African (AFR) | African Ancestry (SW US), African Caribbean, Luhya (Kenya), Mende (Sierra Leone), Gambian Mandinka, Esan (S Nigeria), Yoruba (Nigeria & Benin) | Not specified | Black or African American |
| Admixed American (AMR) | Puerto Rican, Peruvian, Colombian, Mexican | Not specified | Hispanic or Latino, American Indian or Alaska Native |
| East Asian (EAS) | Northern Chinese, Southern Chinese, Vietnamese, Dai (SW China), Japanese | Not specified | Asian |
| European (EUR) | English & Scottish, Italian, Finnish, N&W European, Iberian (Spain & Portugal) | Not specified | White or Caucasian |
| South Asian (SAS) | Punjabi (Pakistan), Bengali, Gujarati (W India), Tamil Nadu-India, Telugu (SE India) | Not specified | Asian |

**Supplemental Table 2.**

3D Health Study participant demographics (N = 166).

| **Variable** | **Completed Survey (N = 166) n (%)** |
| --- | --- |
| **Gender** |  |
| Man | 64 (38.6%) |
| Woman | 101 (60.8%) |
| Non-binary | 0 (0.0%) |
| Prefer not to answer | 1 (0.6%) |
| **Age** |  |
| Below 30 | 7 (4.2%) |
| 30–45 | 49 (29.5%) |
| 45–60 | 27 (16.3%) |
| 60 or above | 82 (49.4%) |
| Prefer not to answer | 1 (0.6%) |
| **Race/Ethnicityᵃ** |  |
| American Indian/Alaska Native | 1 (0.6%) |
| Asian | 34 (20.5%) |
| Black/African American | 4 (2.4%) |
| Hispanic/Latino | 9 (5.4%) |
| Native Hawaiian/Pacific Islander | 0 (0.0%) |
| White | 124 (74.7%) |
| Other | 10 (6.0%) |
| **Had Prior Genetic Ancestry Testing** |  |
| Yes | 74 (44.6%) |
| No | 89 (53.6%) |
| Missing | 3 (1.8%) |

*ᵃ Participants were allowed to select more than one race/ethnicity.*

**Supplemental Table 3.**

UCSF provider demographics (N = 31).

| **Variable** | **Completed Survey (N = 31) n (%)** |
| --- | --- |
| **Gender** |  |
| Man | 5 (16.1%) |
| Woman | 25 (80.6%) |
| Non-binary | 0 (0.0%) |
| Prefer not to answer | 1 (3.2%) |
| **Age** |  |
| Below 30 | 0 (0.0%) |
| 30–45 | 18 (58.1%) |
| 45–60 | 11 (35.5%) |
| 60 or above | 2 (6.5%) |
| Prefer not to answer | 0 (0.0%) |
| **Race/Ethnicityᵃ** |  |
| American Indian/Alaska Native | 0 (0.0%) |
| Asian | 9 (29.0%) |
| Black/African American | 2 (6.5%) |
| Hispanic/Latino | 2 (6.5%) |
| Native Hawaiian/Pacific Islander | 0 (0.0%) |
| White | 21 (67.7%) |
| Other | 3 (9.7%) |
| **Specialty** |  |
| Internal Medicine | 12 (38.7%) |
| Family Medicine | 2 (6.5%) |
| Genetics | 1 (3.2%) |
| Genetic Counseling | 14 (45.2%) |
| Other | 2 (6.5%) |
| **Had Prior Genetic Ancestry Testing** |  |
| Yes | 5 (16.1%) |
| No | 26 (83.9%) |

*ᵃ Participants were allowed to select more than one race/ethnicity.*

**Supplemental Table 4.**

Concordance between genetic ancestry calculations. Genetic ancestry results from two commercial genetic testing providers and two in-house analyses were compared using genetic samples in the UCSF 3D Health Study. Agreement was determined by the percentage of overlapping ancestry from each ancestry location. P-values were determined using a Wilcoxon Rank Sum test. *p < 0.05; **p < 0.005.

| **Comparison** | **Min** | **Median** | **Max** | **Mean** | **p (Afr)** | **p (As)** | **p (Adm)** |
| --- | --- | --- | --- | --- | --- | --- | --- |
| ***Ancestry 1 vs. Ancestry 3 (n = 460)*** | | | | | | | |
| Overall | 0.00% | 54.65% | 94.91% | 57.00% |  |  |  |
| European (333) | 26.00% | 53.41% | 78.03% | 53.34% | 6.0e-5** | 6.6e-22** | 0.63 |
| African (9) | 53.57% | 79.99% | 94.91% | 76.18% |  | 0.37 | 9.8e-4** |
| Asian (75) | 36.45% | 78.26% | 89.94% | 72.90% |  |  | 5.7e-6** |
| Admixed American (18) | 0.00% | 53.34% | 72.78% | 52.52% |  |  |  |
| ***Ancestry 1 vs. Ancestry 4 (n = 460)*** | | | | | | | |
| Overall | 0.00% | 54.79% | 95.21% | 57.26% |  |  |  |
| European (333) | 32.27% | 52.96% | 79.67% | 53.12% | 2.5e-3** | 1.6e-29** | 1.3e-3** |
| African (9) | 47.92% | 79.99% | 95.21% | 73.69% |  | 0.75 | 0.076 |
| Asian (75) | 38.66% | 78.67% | 89.94% | 74.53% |  |  | 9.4e-6** |
| Admixed American (18) | 0.00% | 62.70% | 74.77% | 58.21% |  |  |  |
| ***Ancestry 2 vs. Ancestry 3 (n = 465)*** | | | | | | | |
| Overall | 33.50% | 99.87% | 100.00% | 95.80% |  |  |  |
| European (337) | 33.50% | 99.90% | 100.00% | 97.24% | 7.5e-4** | 4.0e-3** | 1.3e-9** |
| African (9) | 92.73% | 94.53% | 97.10% | 94.97% |  | 2.1e-4** | 6.0e-4** |
| Asian (74) | 78.60% | 100.00% | 100.00% | 97.62% |  |  | 3.7e-10** |
| Admixed American (18) | 38.72% | 65.15% | 100.00% | 66.83% |  |  |  |
| ***Ancestry 2 vs. Ancestry 4 (n = 465)*** | | | | | | | |
| Overall | 58.68% | 100.00% | 100.00% | 95.70% |  |  |  |
| European (337) | 58.68% | 100.00% | 100.00% | 97.33% | 3.5e-5** | 2.4e-15** | 2.3e-12** |
| African (9) | 83.13% | 95.52% | 98.62% | 94.37% |  | 0.040* | 3.1e-3** |
| Asian (74) | 71.67% | 97.97% | 100.00% | 94.65% |  |  | 6.3e-6** |
| Admixed American (18) | 58.72% | 75.65% | 100.00% | 78.32% |  |  |  |
| ***Ancestry 3 vs. Ancestry 4 (n = 474)*** | | | | | | | |
| Overall | 58.77% | 99.41% | 100.00% | 97.05% |  |  |  |
| European (344) | 71.90% | 99.72% | 100.00% | 98.76% | 2.3e-4** | 1.3e-13** | 6.0e-9** |
| African (9) | 81.30% | 98.48% | 99.01% | 96.39% |  | 0.90 | 6.2e-3* |
| Asian (76) | 62.90% | 97.91% | 100.00% | 93.54% |  |  | 1.7e-3** |
| Admixed American (18) | 58.77% | 89.40% | 100.00% | 86.08% |  |  |  |

**Supplemental Table 5.**

Pairwise comparisons of the concordance of the four ancestry sources when compared to Apex-reported ancestry. McNemar’s test was used on each pairwise comparison. *p < 0.05; **p < 0.005.

| **Comparison** | **50% Threshold** | **75% Threshold** | **90% Threshold** |
| --- | --- | --- | --- |
| Ancestry 1 / 2 | 5.0e-24** | 1.7e-70** | 9.6e-74** |
| Ancestry 1 / 3 | 1.4e-21** | 6.4e-71** | 3.8e-81** |
| Ancestry 1 / 4 | 1.5e-22** | 1.4e-71** | 3.1e-82** |
| Ancestry 2 / 3 | 0.21 | >0.99 | 4.0e-7** |
| Ancestry 2 / 4 | 0.23 | 0.45 | 1.1e-8** |
| Ancestry 3 / 4 | >0.99 | 0.131 | 0.041* |

**Supplemental Table 6.**

Provider perspectives on the integration of genetic ancestry into the electronic health record. Responses were totaled for the overall responses from all providers and then calculated based on the provider’s self-identified race/ethnicity. Providers who selected more than one race/ethnicity that did not fit into one of the general ancestry categories were placed into Other. A Fisher’s Exact test was used to determine if there was a statistically significant difference in responses between ancestry groups.

|  | **Strongly Disagree** | **Somewhat Disagree** | **Neutral** | **Somewhat Agree** | **Strongly Agree** | **p-value** |
| --- | --- | --- | --- | --- | --- | --- |
| **Overall (N = 31)** | **2 (6.5%)** | **6 (19.4%)** | **16 (51.6%)** | **7 (22.6%)** | **0 (0.0%)** |  |
| European (n = 18) | 1 (5.6%) | 4 (22.2%) | 8 (44.4%) | 5 (27.8%) | 0 (0.0%) |  |
| African (n = 1) | 0 (0.0%) | 0 (0.0%) | 1 (100.0%) | 0 (0.0%) | 0 (0.0%) |  |
| Asian (n = 7) | 1 (14.3%) | 1 (14.3%) | 4 (57.1%) | 1 (14.3%) | 0 (0.0%) | 0.99 |
| Adm. American (n = 1) | 0 (0.0%) | 0 (0.0%) | 1 (100.0%) | 0 (0.0%) | 0 (0.0%) |  |
| Other (n = 4) | 0 (0.0%) | 1 (25.0%) | 2 (50.0%) | 1 (25.0%) | 0 (0.0%) |  |

**Supplemental Table 7.**

**SIRE-stratified participant responses to questions regarding the impact of genetic ancestry results on identity (N = 166).** *Responses from Table 3 stratified by self-identified race/ethnicity. Participants who selected more than one race/ethnicity that did not fit into one of the general ancestry categories were placed into Other. A Fisher’s Exact test was used to determine if there was a statistically significant difference in responses between ancestry groups. Questions based on Rubanovich et al. 2021.*

**Were your ancestry test results surprising or unexpected?** *(p = 0.0065*)*

| **SIRE Group** | **Yes** | **No** | **Somewhat/Maybe** | **Missing** |
| --- | --- | --- | --- | --- |
| White/European (n=115) | 40 (34.8%) | 36 (31.3%) | 38 (33.0%) | 1 (0.9%) |
| Black/African American (n=3) | 0 (0.0%) | 0 (0.0%) | 3 (100.0%) | 0 (0.0%) |
| Asian (n=29) | 6 (20.7%) | 15 (51.7%) | 7 (24.1%) | 1 (3.4%) |
| Mixed American (n=6) | 2 (33.3%) | 1 (16.7%) | 3 (50.0%) | 0 (0.0%) |
| Other (n=13) | 5 (38.5%) | 0 (0.0%) | 8 (61.5%) | 0 (0.0%) |

**Were your ancestry test results undesired or distressing?** *(p = 0.72)*

| **SIRE Group** | **Yes** | **No** | **Somewhat/Maybe** | **Missing** |
| --- | --- | --- | --- | --- |
| White/European (n=115) | 1 (0.9%) | 110 (95.7%) | 3 (2.6%) | 1 (0.9%) |
| Black/African American (n=3) | 0 (0.0%) | 3 (100.0%) | 0 (0.0%) | 0 (0.0%) |
| Asian (n=29) | 1 (3.4%) | 26 (89.7%) | 0 (0.0%) | 2 (6.9%) |
| Mixed American (n=6) | 0 (0.0%) | 6 (100.0%) | 0 (0.0%) | 0 (0.0%) |
| Other (n=13) | 0 (0.0%) | 13 (100.0%) | 0 (0.0%) | 0 (0.0%) |

**Do your ancestry test results change your perceptions of your cultural roots?** *(p = 0.076)*

| **SIRE Group** | **Yes** | **No** | **Somewhat/Maybe** | **Missing** |
| --- | --- | --- | --- | --- |
| White/European (n=115) | 10 (8.7%) | 80 (69.6%) | 23 (20.0%) | 2 (1.7%) |
| Black/African American (n=3) | 0 (0.0%) | 0 (0.0%) | 3 (100.0%) | 0 (0.0%) |
| Asian (n=29) | 4 (13.8%) | 19 (65.5%) | 5 (17.2%) | 1 (3.4%) |
| Mixed American (n=6) | 1 (16.7%) | 3 (50.0%) | 2 (33.3%) | 0 (0.0%) |
| Other (n=13) | 3 (23.1%) | 7 (53.8%) | 3 (23.1%) | 0 (0.0%) |

**Do your ancestry test results change the likelihood that you would travel to certain parts of the world?** *(p = 0.57)*

| **SIRE Group** | **Yes** | **No** | **Somewhat/Maybe** | **Missing** |
| --- | --- | --- | --- | --- |
| White/European (n=115) | 3 (2.6%) | 103 (89.6%) | 6 (5.2%) | 3 (2.6%) |
| Black/African American (n=3) | 0 (0.0%) | 3 (100.0%) | 0 (0.0%) | 0 (0.0%) |
| Asian (n=29) | 1 (3.4%) | 26 (89.7%) | 1 (3.4%) | 1 (3.4%) |
| Mixed American (n=6) | 0 (0.0%) | 5 (83.3%) | 1 (16.7%) | 0 (0.0%) |
| Other (n=13) | 1 (7.7%) | 11 (84.6%) | 1 (7.7%) | 0 (0.0%) |

**Do your ancestry test results change how you view certain cultures or world regions?** *(p = 0.13)*

| **SIRE Group** | **Yes** | **No** | **Somewhat/Maybe** | **Missing** |
| --- | --- | --- | --- | --- |
| White/European (n=115) | 1 (0.9%) | 109 (94.8%) | 3 (2.6%) | 2 (1.7%) |
| Black/African American (n=3) | 1 (33.3%) | 2 (66.7%) | 0 (0.0%) | 0 (0.0%) |
| Asian (n=29) | 1 (3.4%) | 25 (86.2%) | 1 (3.4%) | 2 (6.9%) |
| Mixed American (n=6) | 0 (0.0%) | 6 (100.0%) | 0 (0.0%) | 0 (0.0%) |
| Other (n=13) | 1 (7.7%) | 12 (92.3%) | 0 (0.0%) | 0 (0.0%) |

**Would you say your ancestry test results have reshaped your personal identity?** *(p = 0.096)*

| **SIRE Group** | **Yes** | **No** | **Somewhat/Maybe** | **Missing** |
| --- | --- | --- | --- | --- |
| White/European (n=115) | 4 (3.5%) | 100 (87.0%) | 10 (8.7%) | 1 (0.9%) |
| Black/African American (n=3) | 0 (0.0%) | 2 (66.7%) | 1 (33.3%) | 0 (0.0%) |
| Asian (n=29) | 3 (10.3%) | 25 (86.2%) | 0 (0.0%) | 1 (3.4%) |
| Mixed American (n=6) | 0 (0.0%) | 5 (83.3%) | 0 (0.0%) | 1 (16.7%) |
| Other (n=13) | 0 (0.0%) | 9 (69.2%) | 3 (23.1%) | 1 (7.7%) |

**Do you plan to share your ancestry test results with your family members?** *(p = 0.33)*

| **SIRE Group** | **Yes** | **No** | **Somewhat/Maybe** | **Missing** |
| --- | --- | --- | --- | --- |
| White/European (n=115) | 88 (76.5%) | 11 (9.6%) | 15 (13.0%) | 1 (0.9%) |
| Black/African American (n=3) | 3 (100.0%) | 0 (0.0%) | 0 (0.0%) | 0 (0.0%) |
| Asian (n=29) | 20 (69.0%) | 4 (13.8%) | 4 (13.8%) | 1 (3.4%) |
| Mixed American (n=6) | 5 (83.3%) | 1 (16.7%) | 0 (0.0%) | 0 (0.0%) |
| Other (n=13) | 6 (46.2%) | 4 (30.8%) | 3 (23.1%) | 0 (0.0%) |

**Will you provide or discuss your ancestry test results with your physician or a healthcare provider?** *(p = 0.061)*

| **SIRE Group** | **Yes** | **No** | **Somewhat/Maybe** | **Missing** |
| --- | --- | --- | --- | --- |
| White/European (n=115) | 24 (20.9%) | 46 (40.0%) | 43 (37.4%) | 2 (1.7%) |
| Black/African American (n=3) | 3 (100.0%) | 0 (0.0%) | 0 (0.0%) | 0 (0.0%) |
| Asian (n=29) | 5 (17.2%) | 12 (41.4%) | 11 (37.9%) | 1 (3.4%) |
| Mixed American (n=6) | 1 (16.7%) | 2 (33.3%) | 3 (50.0%) | 0 (0.0%) |
| Other (n=13) | 1 (7.7%) | 10 (76.9%) | 2 (15.4%) | 0 (0.0%) |

**If you had genetic ancestry testing previously, do you perceive your current results to be different?** *(p = 0.0066*)*

| **SIRE Group** | **Yes** | **No** | **Somewhat** | **N/A / Missing** |
| --- | --- | --- | --- | --- |
| White/European (n=115) | 25 (21.7%) | 21 (18.3%) | 12 (10.4%) | 49 (42.6%) / 8 (7.0%) |
| Black/African American (n=3) | 0 (0.0%) | 0 (0.0%) | 3 (100.0%) | 0 (0.0%) / 0 (0.0%) |
| Asian (n=29) | 5 (17.2%) | 5 (17.2%) | 2 (6.9%) | 13 (44.8%) / 4 (13.8%) |
| Mixed American (n=6) | 5 (83.3%) | 0 (0.0%) | 0 (0.0%) | 1 (16.7%) / 0 (0.0%) |
| Other (n=13) | 2 (15.4%) | 0 (0.0%) | 1 (7.7%) | 10 (76.9%) / 0 (0.0%) |

**Does the experience of undergoing genetic ancestry testing make you more or less likely to have other genetic tests?** *(p = 0.016*)*

| **SIRE Group** | **More Likely** | **Less Likely** | **No Change** | **Missing** |
| --- | --- | --- | --- | --- |
| White/European (n=115) | 38 (33.0%) | 11 (9.6%) | 64 (55.7%) | 2 (1.7%) |
| Black/African American (n=3) | 3 (100.0%) | 0 (0.0%) | 0 (0.0%) | 0 (0.0%) |
| Asian (n=29) | 9 (31.0%) | 0 (0.0%) | 19 (65.5%) | 1 (3.4%) |
| Mixed American (n=6) | 2 (33.3%) | 1 (16.7%) | 3 (50.0%) | 0 (0.0%) |
| Other (n=13) | 1 (7.7%) | 4 (30.8%) | 8 (61.5%) | 0 (0.0%) |

**Supplemental Table 8.**

*Concordance between genetic ancestry results and self-identified race/ethnicity (SIRE), stratified by SIRE group. Genetic ancestry results from two commercial genetic testing companies (Ancestry 1 and Ancestry 2) and two supplemental ancestry calculations (Ancestry 3 and Ancestry 4) were compared to Apex-reported SIRE at three concordance thresholds (≥50%, ≥75%, ≥90%). Values are percent concordant (n concordant/n total). Small subgroup sizes for African (n=9) and Mixed American (n=18) participants limit interpretation of within-group differences.*

**≥50% Concordance Threshold**

| **SIRE Group** | **Ancestry 1** | **Ancestry 2** | **Ancestry 3** | **Ancestry 4** |
| --- | --- | --- | --- | --- |
| European | 67.48% (220/326) | 97.92% (330/337) | 98.51% (332/337) | 98.81% (333/337) |
| African | 77.78% (7/9) | 66.67% (6/9) | 66.67% (6/9) | 66.67% (6/9) |
| Asian | 93.15% (68/73) | 95.95% (71/74) | 98.65% (73/74) | 94.59% (70/74) |
| Mixed American | 16.67% (3/18) | 55.56% (10/18) | 0.00% (0/18) | 16.67% (3/18) |
| **Overall** | **69.63% (298/428)** | **94.78% (418/441)** | **93.42% (412/441)** | **93.65% (413/441)** |

**≥75% Concordance Threshold**

| **SIRE Group** | **Ancestry 1** | **Ancestry 2** | **Ancestry 3** | **Ancestry 4** |
| --- | --- | --- | --- | --- |
| European | 0% (0/326) | 96.14% (324/337) | 96.44% (325/337) | 97.33% (328/337) |
| African | 44.44% (4/9) | 55.56% (5/9) | 55.56% (5/9) | 55.56% (5/9) |
| Asian | 86.30% (63/73) | 90.54% (67/74) | 91.89% (68/74) | 91.89% (68/74) |
| Mixed American | 0.00% (0/18) | 16.67% (3/18) | 0.00% (0/18) | 5.56% (1/18) |
| **Overall** | **15.65% (67/428)** | **90.70% (400/441)** | **90.48% (399/441)** | **91.38% (403/441)** |

**≥90% Concordance Threshold**

| **SIRE Group** | **Ancestry 1** | **Ancestry 2** | **Ancestry 3** | **Ancestry 4** |
| --- | --- | --- | --- | --- |
| European | 0% (0/326) | 83.38% (281/337) | 94.96% (320/337) | 96.74% (326/337) |
| African | 22.22% (2/9) | 33.33% (3/9) | 22.22% (2/9) | 22.22% (2/9) |
| Asian | 12.33% (9/73) | 87.84% (65/74) | 87.84% (65/74) | 87.84% (65/74) |
| Mixed American | 0.00% (0/18) | 16.67% (3/18) | 0.00% (0/18) | 0.00% (0/18) |
| **Overall** | **2.57% (11/428)** | **80.05% (353/441)** | **87.98% (388/441)** | **89.34% (394/441)** |

*Note: Ancestry 1 and Ancestry 2 are commercial genetic testing companies. Ancestry 3 and Ancestry 4 are in-house analyses using reference panels from the 1,000 Genomes Project and 100,000 Genomes Project, respectively. SIRE groups are based on the primary Apex-reported race/ethnicity; participants selecting more than one classifiable category had proportions evenly distributed. Participants with only ND categories (Mixed, Declined, Not Available, or Native Hawaiian/Other Pacific Islander) were excluded from concordance analysis.*

**Supplemental Figure 1.**

Flowchart of the 3D Health Study participants active and recontacted to receive genetic ancestry reports and complete the additional ancestry perspectives survey.

**Supplemental Figure 2.**

3D Health participant concerns about the possible unintended outcomes of genetic ancestry testing.

**Supplemental Figure 3.**

3D Health participant concerns on the use of genetic ancestry reports in the electronic health record.
